# Dynamic stability of the euler nanobeam subjected to inertial moving nanoparticles based on the nonlocal strain gradient theory

**DOI:** 10.1016/j.heliyon.2024.e30231

**Published:** 2024-04-27

**Authors:** Mohammad Hashemian, Dheyaa J. Jasim, S. Mohammad Sajadi, Rahman Khanahmadi, Mostafa Pirmoradian, Soheil Salahshour

**Affiliations:** a Department of Mechanical Engineering, Khomeinishahr Branch, Islamic Azad University, Khomeinishahr, Iran; b Department of Petroleum Engineering, Al-Amarah University College, Maysan, Iraq; c Department of Nutrition, Cihan University-Erbil, Kurdistan Region, Iraq; d Department of Mechanical Engineering, Isfahan University of Technology, Isfahan, 84156-83111, Iran; e Faculty of Engineering and Natural Sciences, Istanbul Okan University, Istanbul, Turkey; f Faculty of Engineering and Natural Sciences, Bahcesehir University, Istanbul, Turkey; g Department of Computer Science and Mathematics, Lebanese American University, Beirut, Lebanon

**Keywords:** Dynamic stability, Moving nanoparticle, Nonlocal strain gradient theory, Hamilton's principle, Surface effect, EBT, IHBM

## Abstract

This research studied the dynamic stability of the Euler-Bernoulli nanobeam considering the nonlocal strain gradient theory (NSGT) and surface effects. The nanobeam rests on the Pasternak foundation and a sequence of inertial nanoparticles passes above the nanobeam continuously at a fixed velocity. Surface effects have been utilized using the Gurtin-Murdoch theory. Final governing equations have been gathered implementing the energy method and Hamilton's principle alongside NSGT. Dynamic instability regions (DIRs) are drawn in the plane of mass-velocity coordinates of nanoparticles based on the incremental harmonic balance method (IHBM). A parametric study shows the effects of NSGT parameters and Pasternak foundation constants on the nanobeam's DIRs. In addition, the results exhibit the importance of 2T-period DIRs in comparison to T-period ones. According to the results, the Winkler spring constant is more effective than the Pasternak shear constant on the DIR movement of nanobeam. So, a 4 times increase of Winkler and Pasternak constants results in 102 % and 10 % of DIR movement towards higher velocity regions, respectively. Furthermore, the effect of increasing nonlocal and material length scale parameters on the DIR movement are in the same order regarding the magnitude but opposite considering the motion direction. Unlike nonlocal parameter, an increase in material length scale parameter shifts the DIR to the more stable region.

**Table:** Nomenclature

Description	Symbol
Middle surface Displacement of the nanobeam along the *x* and *z* axes	u, w
Displacement of components of an arbitrary point	$U_{x},U_{Y},U_{z}$
Length, width, and height of the nanobeam	L, b, h
Time	t
Bulk Young's modulus	E
Nanobeam Poisson's ratio	υ
Winker spring constant	$K_{w}$
Pasternak shear constant	$K_{G}$
Nanoparticle mass	m
Nonlocal parameter	ea
Material length parameter	l
Nanoparticle velocity	$V_{m}$
Surface Young's modulus	$E^{s}$
Surface shear residual stress	$\tau^{s}$
Normal strain	$\varepsilon_{xx}$
Bulk density	ρ
Shear density	$\rho^{s}$
The axial component of the bulk stress	$\sigma_{xx}$
The axial component of the surface stress	$\sigma_{xx}^{s}$

## 1 Introduction

Nanotechnology exhibited unique mechanical properties at the nanoscale, enabling precise control and manipulation of materials for various applications. These characteristics included enhanced strength, flexibility, and surface area-to-volume ratio. Classical theories of mechanics, such as continuum mechanics, were not entirely applicable in nanotechnology due to the size and scale of nanomaterials. At the nanoscale, the behavior of materials was governed by quantum mechanics, and classical theories failed to accurately describe the phenomena observed. As a result, new theories have emerged to explain the mechanical behavior of nanomaterials. These included nonlocal elasticity theory [1], couple stress theory [2], strain gradient theory [3], nonlocal strain gradient theory [4], etc. These new theories took into consideration the effects of surface energy, nonlocal interactions, and higher-order gradients that were not present in classical theories.

Ansari and Sahmani [5] conducted the bending and buckling analyses of nanobeam considering the significance of the surface layer. It was noted that as the aspect ratio increased, the impact of surface stress on the bending behavior of nanobeams became more pronounced. The nonlinear lateral oscillation and instability of single-walled boron nitride nanotubes carrying a viscous fluid were reviewed by Arani et al. [6] considering the nonlocal piezoelasticity and multiple-scale methods. Hosseini-Hashemi et al. [7] utilized the Surface theory for nonlinear nonlocal free vibration evaluation of functionally graded (FG) nanobeams. The results showed that at low mode numbers, surface effects had a strong impact. However, as the mode number increased, the nonlocal parameter became more influential.

In comparison to Eringen's theory, the NSGT held significance as it incorporated both nonlocal effects and strain gradient effects, enabling a more comprehensive understanding of the mechanical behavior of nanomaterials [8,9]. Lu et al. [10] analyzed the vibration of nanobeams according to NSGT. Results depicted that the predicted natural frequency based on the NSGT was lower than one in the local strain gradient theory. Wang et al. [11] considering the axial motion of the nanobeams, checked out nonlinear free vibrations and flutter instability based on the NSGT. Based on the results, the flutter critical velocity dropped as the nonlocal parameter rose and increased as the material characteristic parameter increased. Ebrahimi and Dabbagh [12] reported the wave dispersion behaviors of sandwich composite nanoplates, taking into account the magnetostriction phenomenon and the influence of scale using the NSGT. Tang et al. [13] examined the Poisson's ratio and thickness effects on the vibration behavior of nanobeams incorporating NSGT. They found NSGT beam demonstrated stiffness-hardening in low-order modes due to the thickness effect but showed stiffness-softening in high-order modes. Shen et al. [14] simulated the transverse vibration of microtubules under combined thermos-mechanical loading based on NSGT. They verified the presence of limits for nonlocal scale and strain gradient parameters. Li et al. [15] modeled the dynamic behavior of a self-powered piezoelectric nanoribbon in the presence of thermal, mechanical, and electrical fields based on NSGT. Results revealed the interaction of multiple physical fields didn't alter how the nonlocal and strain gradient characteristic parameters affected the frequencies of self-powered nanoribbons.

The movement of nanoparticles on nanostructures had diverse applications. It could be utilized in nanoscale drug delivery systems for targeted and controlled release of therapeutic agents. It also found applications in nanosensors for detecting and monitoring environmental pollutants or biological analyses. Furthermore, the directed movement of nanoparticles on nanostructures could be harnessed in nanoelectronics for creating high-density data storage devices or nanoscale circuitry [16,17]. Kiani and Mehri [18] carried out the nonlocal dynamic analysis of nanotubes under a mobile nanoparticle excitation considering various beam models. In other research, the nonlocal forced vibration of a single-walled carbon nanotube (SWCNT) influenced by a passing load was examined by Şimşek [19]. Chang [20] addressed the stochastic finite element method (FEM) to examine the statistical dynamic characteristics of fluid-conveying double-walled carbon nanotubes (DWCNTs) exposed to a load in motion. Hashemi and Khaniki [21] perused the nonlocal dynamic analysis of coupled Euler-Bernoulli nanobeams in a multiple nano beam system (MNBS) subjected to a mobile nanoparticle. According to the results, changes in the nonlocal parameter had the greatest impact on the top layer of MNBS, where the nanoparticle was in motion. Ejabati and Fallah [22] explored the influence of air drag on the nonlocal dynamic analysis of Mindlin nanoplate subjected to a moving nanoparticle. This study employed the mesh-free finite volume method to investigate this effect. Yu et al. [23] conducted the nonlocal transverse vibrations of single-layered membranes composed of SWCNTs under the action of mobile nanoparticles in each nanotube. The characteristics of nanoparticles such as velocity, mass, inertia, and lag on the deflection of the mentioned MNBS were examined in this survey.

Dynamic instability pertains to the behavior of mechanical and elastic systems when subjected to time-varying loads, particularly those of a periodic nature. Unlike forced vibration problems, in dynamic stability surveys, time-dependent excitation terms appeared as the coefficients of the homogenous governing differential equations [24]. Ke et al. [25] studied the dynamic stability of the FG microbeams according to the modified couple stress theory and Timoshenko beam theory (TBT). The impact of size on the dynamic stability properties was noteworthy only when the microbeam's thickness closely matched the material length scale parameter. Li et al. [26] implemented a method of multiple scales to analyze nonlocal free vibration and parametric stability of a nanobeam that experiences varying axial forces. Huang et al. [27] evaluated the nonlocal dynamic stability of nanobeams based on Euler-Bernoulli beam theory (EBT) considering Bolotin's method. Based on the results, the critical excitation frequencies and their bandwidth decreased significantly as the nonlocal parameter increased.

Lau et al. [28] proposed IHBM for the parametric study of linear and nonlinear columns. Based on the results, this approach easily handled minor parameter adjustments and was convenient for computer programming. Pirmoradian et al. [29] examined the dynamic instability of DWCNTs surrounded by an elastic medium subjected to parametric excitation caused by the sequential moving nanoparticles. Results showed taking into account the van der Waals force results in moving the unstable region to higher frequencies for the passage of nanoparticles.

According to the knowledge of the authors, the parametric instability of an Euler-Bernoulli nanobeam considering NSGT and surface effects under a sequence of inertial moving nanoparticles has not been studied. Governing equations have been extracted using the energy method and Hamilton's principle. Nanobeam DIRs have been plotted in the plane of dimensionless mass-velocity coordinates employing IHBM and the effect of various parameters has been analyzed.

## 2 Problem definition

**Fig. 1:**
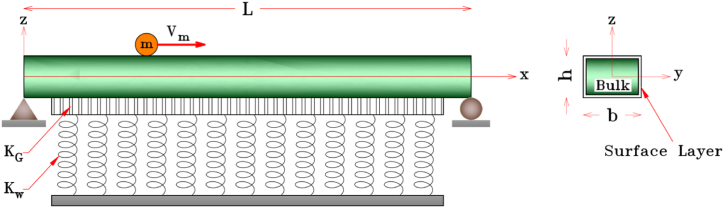
Schematic of a rested nanobeam under passing nanoparticles.

As seen in Fig. 1, consider a nanobeam with dimensions L, b, and h as the length, width, and height of the nanobeam, respectively. The nanobeam rests on the Pasternak foundation in which $K_{w}$ and $K_{G}$ are Winker spring and Pasternak shear constants of the foundation, respectively. Nanoparticles also pass over the nanobeam continuously with a mass of m and a constant velocity of $V_{m}$. It is assumed that as soon as one nanoparticle departs from the nanobeam, the subsequent one takes its place and continues the process, and the moving nanoparticle is in contact with the nanobeam throughout its motion.

## 3 Displacement and strain fields

Utilizing EBT, the nanobeam's displacement field can be stated as [30].

(1) $$\begin{array}{l} U_{x}\left(x,z,t\right)=u\left(x,t\right)-z\frac{\partial w\left(x,t\right)}{\partial x}\text{,}\\ U_{y}\left(x,z,t\right)=0\text{,}\\ U_{z}\left(x,z,t\right)=w\left(x,t\right)\text{,} \end{array}$$

where $U_{x},U_{y}$ and $U_{z}$, are the displacement components at any point along the x,y and z directions, sequentially. u and w denote the middle surface displacement of the nanobeam along the x and z axes, respectively and t determines the time. The strain field can be derived according to kinematic relation as [30]

(2) $$\varepsilon_{xx}=\frac{\partial u\left(x,t\right)}{\partial x}-z\frac{\partial^{2}w\left(x,t\right)}{\partial^{2}x},\varepsilon_{yy}=\varepsilon_{zz}=\gamma_{xy}=\gamma_{yz}=\gamma_{xz}=0..$$

## 4 Deriving governing equations

Energy methods can be categorized into two branches. Principles of virtual force, virtual displacement, minimum total potential energy, and minimum total complementary energy as the first category can be used to derive governing equations and boundary conditions of a continuous system. On the other hand, Castigliano's methods, unit dummy load, and unit dummy displacement can be accounted as the second category in which desired points are examined.

In the current study, Hamilton's principle as the generalized form of the virtual displacement method is used to derive governing equations and boundary conditions. In the next, strain energy, kinetic energy, and potential energy are discussed for the nanobeam.

### 4.1 The kinetic energy of the nanobeam

Considering the time derivative of Eq. (1), total kinetic energy including bulk kinetic energy $\left(K^{b}\right)$ and surface kinetic energy $\left(K^{s}\right)$ and its variation form can be derived as [31]

(3) $$\begin{array}{l} K^{b}+K^{s}=\frac{1}{2}\rho \int_{0}^{L}\int_{A}V_{b}^{2}dA\;dx+\frac{1}{2}\rho_{s}\int_{0}^{L}\oint V_{b}^{2}dS\;dx\text{,} \end{array}$$

(4) $$\int_{0}^{t}\left(\delta K^{b}+\delta K^{s}\right)dt=-\int_{0}^{t}\int_{0}^{L}\left[\left(\rho bh+2\rho^{s}\left(b+h\right)\right)\left(\frac{\partial^{2}u}{\partial t^{2}}\right)\delta u\right]dt--\int_{0}^{t}\int_{0}^{L}\left[\left(\rho bh+2\rho^{s}\left(b+h\right)\right)\left(\frac{\partial^{2}w}{\partial t^{2}}\right)\delta w\right]dt\text{,}$$

where ρ and $\rho^{s}$ indicate bulk and surface density, respectively. $V_{b}$ indicates the nanobeam velocity, A and S refer to the cross-sectional area and perimeter of the nanobeam and b,s superscripts refer to bulk and surface properties, respectively.

### 4.2 The potential energy of the Pasternak foundation

The variation form of the Pasternak foundation potential energy is calculated as follows [32].

(5) $$\delta V=-\int_{0}^{L}F_{m}\delta w\;b\;dx\text{,}$$

(6) $$F_{m}=K_{w}w-K_{G}\frac{\partial^{2}w}{\partial x^{2}}\text{,}$$

where $K_{w},K_{G}$ as illustrated in Fig. 1 are the Winkler and Pasternak constants of the foundation, respectively.

### 4.3 Energies of the nanoparticles

The variation of nanoparticle potential energy due to gravitational effect can be expressed as

(7) $$\int_{0}^{t}\delta PE_{mass}dt=\int_{0}^{t}\int_{0}^{L}mg\delta w\bar{\delta }\left(x-V_{m}\;t\right)dx\;dt\text{,}$$

where g represents gravitational acceleration and $\bar{\delta }\left(x-V_{m}t\right)$ denotes the Dirac delta function. The kinetic energy variation of the nanoparticle is derived as

(8) $$\begin{array}{l} \int_{0}^{t}\delta KE_{mass}dt=-\int_{0}^{t}\int_{0}^{L}m\left[\frac{\partial }{\partial t}\left(\frac{\partial u}{\partial t}\bar{\delta }\left(x-x_{m}\right)\right)\delta u+\frac{\partial }{\partial t}\left(\frac{\partial w}{\partial t}\bar{\delta }\left(x-x_{m}\right)\right)\delta w\right]dxdt-\\ \int_{0}^{t}\int_{0}^{L}m\left[\frac{\partial }{\partial t}\left(V_{m}\bar{\delta }\left(x-x_{m}\right)\right)\delta u+V_{m}\frac{\partial }{\partial t}\left(\frac{\partial w}{\partial x}\bar{\delta }\left(x-x_{m}\right)\right)\delta w\right]dxdt-\\ \int_{0}^{t}\int_{0}^{L}m\left[V_{m}\frac{\partial }{\partial x}\left(\frac{\partial w}{\partial t}\bar{\delta }\left(x-x_{m}\right)\right)\delta w\right]dxdt\text{.} \end{array}$$

### 4.4 Strain energy based on NSGT and surface effects

The NSGT, which is one of the useful theories of non-classical continuums mechanics, can be stated as follows [4].

(9) $$\tilde{t}=\tilde{\sigma }-\nabla {\tilde{\sigma }}^{\left(1\right)}\text{,}$$

where $\tilde{\sigma }$ and ${\tilde{\sigma }}^{\left(1\right)}$ are the classical and the higher-order stress tensors, respectively

(10) $$\tilde{\sigma }=\int_{V}\alpha_{0}\left(X^{\prime },X,e_{0}a\right)\tilde{C}:\tilde{\varepsilon^{\prime }}dV^{\prime }\text{,}$$

(11) $${\tilde{\sigma }}^{\left(1\right)}=l^{2}\int_{V}\alpha_{1}\left(X^{\prime },X,e_{1}a\right)\tilde{C}:\nabla \tilde{\varepsilon^{\prime }}dV^{\prime }\text{,}$$

where l, $e_{0}a$ and $e_{1}a$ are the NSGT length scale parameters that focus on the strain gradient (l), and lower and higher nonlocal stresses $\left(e_{0}a,e_{1}a\right)$. In addition, $\tilde{C}$ is the fourth-order elasticity tensor and $\tilde{\varepsilon^{\prime }}$ and $\nabla \tilde{\varepsilon^{\prime }}$ denote strain and strain gradient tensors, respectively. $\alpha_{0}$ and $\alpha_{1}$ are two attenuation functions that satisfy the conditions given by Eringen. The constitutive equation according to NSGT can be simplified as

(12) $$\left[1-{\left(ea\right)}^{2}\nabla^{2}\right]\tilde{t}=\left(1-l_{0}^{2}\nabla^{2}\right)\tilde{C}:\tilde{\varepsilon }\text{.}$$

If l=0, the NSGT constitutive equation simplifies to the related nonlocal elasticity theory equation.

Strain energy variation according to EBT nanobeam can be calculated as Eq. (13),

(13) $$\delta U^{b}=\int_{V}\left(\sigma_{xx}\delta \varepsilon_{xx}+\sigma_{xx}^{\left(1\right)}\nabla \delta \varepsilon_{xx}\right)dV\text{.}$$

In Eq. (14), the axial stress is calculated using the following equations,

(14) $$\sigma_{xx}=E\varepsilon_{xx}+\upsilon \sigma_{zz}\text{,}$$

where υ is the Poisson's ratio. According to Gurtin-Murdoch theory [33]. In Eq. (14), the value of $\sigma_{zz}$ is as follows

(15) $$\sigma_{zz}=\frac{2z}{h}\left(\tau^{s}\frac{\partial^{2}w}{\partial x^{2}}-\rho^{s}\frac{\partial^{2}w}{\partial t^{2}}\right)\text{,}$$

where $\tau^{s}$ is the residual surface stress.

Finally, the variational relation of the bulk strain energy results as follows

(16) $$\delta U^{b}=-\int_{0}^{L}\left(\frac{\partial N}{\partial x}\delta u+\frac{\partial^{2}M}{\partial x^{2}}\delta w+\frac{\partial S_{1}}{\partial x}\delta u+\frac{\partial^{2}P_{1}}{\partial x^{2}}\delta w\right)dx\text{,}$$

where stress resultants (N and M), $S_{1}$ and $P_{1}$ are

(17) $$N=\int_{A}{\tilde{t}}_{xx}dA,M=\int_{A}{\tilde{t}}_{xx}zdA\text{,}$$

(18) $$S_{1}=\int_{A}\frac{2\upsilon z}{h}\left(\tau^{s}\frac{\partial^{2}w}{\partial x^{2}}-\rho^{s}\frac{\partial^{2}w}{\partial t^{2}}\right)\;dA,P_{1}=\int_{A}\frac{2\upsilon z^{2}}{h}\left(\tau^{s}\frac{\partial^{2}w}{\partial x^{2}}-\rho^{s}\frac{\partial^{2}w}{\partial t^{2}}\right)\;dA\text{.}$$

Also, the strain energy of the surface layer is calculated according to the Gurtin–Murdoch surface theory [34,35],

(19) $$U^{s}=\frac{1}{2}\int_{0}^{L}\left(\oint \sigma_{xx}^{s}\varepsilon_{xx}\right)ds\;dx\text{,}$$

where the axial stress of the surface of the nanobeam is obtained in Eq. (20)

(20) $$\sigma_{xx}^{s}=\tau^{s}+\left(2\mu^{s}+\lambda^{s}\right)\left(\frac{\partial u}{\partial x}-z\frac{\partial^{2}w}{\partial x^{2}}\right)\text{,}$$

where $\mu^{s}$ and $\lambda^{s}$ are surface Lame's constants which are related to surface elasticity modulus $\left(E^{s}\right)$ as

(21) $$\mu^{s}=\frac{E^{s}}{2\left(1+\upsilon \right)},\lambda^{s}=\frac{E^{s}\upsilon }{\left(1+\upsilon \right)\left(1-2\upsilon \right)}\text{.}$$

Finally, the variational configuration of the surface strain energy of the nanobeam results in Eq. (22),

(22) $$\delta U^{s}=-\int_{0}^{L}\left(\frac{\partial N^{s}}{\partial x}\delta u+\frac{\partial^{2}M^{s}}{\partial x^{2}}\delta w\right)dx\text{,}$$

where surface stress resultants are defined as

(23) $$N^{s}=\oint_{A}\sigma_{xx}^{s}ds,M^{s}=\oint_{A}\sigma_{xx}^{s}z\;ds\text{.}$$

### 4.5 Hamilton's principle

Like the virtual displacement method in static problems, Hamilton's principle can be implemented in dynamic ones to derive governing equations [6]. This method for continuous systems can be stated as [36].

(24) $$\int_{0}^{t}\delta L\;dt=0\text{,}$$

(25) $$\int_{0}^{t}\left(\delta U^{b}+\delta U^{s}+\delta PE_{mass}-\delta K^{b}-\delta K^{s}-\delta KE_{mass}-\delta V\right)\;dt=0\text{.}$$

By equating the coefficients of δu and δw in Eq. (25) to zero, classical governing equations can be derived as follows,

(26) $$\begin{array}{l} \delta u\text{:}-\frac{\partial N}{\partial x}-\frac{\partial S_{1}}{\partial x}-\frac{\partial N^{s}}{\partial x}+\left[\rho bh+2\rho^{s}\left(b+h\right)\right]\left(\frac{\partial^{2}u}{\partial t^{2}}\right)+m\frac{\partial^{2}u}{\partial t^{2}}\bar{\delta }\left(x-x_{m}\right)+\\ m\frac{\partial u}{\partial t}\frac{\partial }{\partial t}\bar{\delta }\left(x-x_{m}\right)+mV_{m}\frac{\partial }{\partial t}\bar{\delta }\left(x-x_{m}\right)=0\text{,} \end{array}$$

(27) $$\begin{array}{l} \delta w\text{:}-\frac{\partial^{2}M}{\partial x^{2}}-\frac{\partial^{2}M^{s}}{\partial x^{2}}-\frac{\partial^{2}P_{1}}{\partial x^{2}}+mg\bar{\delta }\left(x-x_{m}\right)+\left[\rho bh+2\rho^{s}\left(b+h\right)\right]\left(\frac{\partial^{2}w}{\partial t^{2}}\right)+\\ m\frac{\partial^{2}w}{\partial t^{2}}\bar{\delta }\left(x-x_{m}\right)+mV_{m}\frac{\partial^{2}w}{\partial x\partial t}\bar{\delta }\left(x-x_{m}\right)+mV_{m}\frac{\partial^{2}w}{\partial x\partial t}\bar{\delta }\left(x-x_{m}\right)+K_{w}bw-\\ K_{G}b\frac{\partial^{2}w}{\partial x^{2}}+m\frac{\partial w}{\partial t}\frac{\partial }{\partial t}\bar{\delta }\left(x-x_{m}\right)+mV_{m}\frac{\partial w}{\partial x}\frac{\partial }{\partial t}\bar{\delta }\left(x-x_{m}\right)+\\ mV_{m}\frac{\partial w}{\partial t}\frac{\partial }{\partial x}\bar{\delta }\left(x-x_{m}\right)=0\text{,} \end{array}$$

and the boundary conditions at x=0,L are

(28) $$\begin{array}{l} N=0\;or\;u=0\text{,}\\ M=0\;or\;w^{\prime }=0\text{,}\\ M^{\prime }=0\;or\;w=0\text{,}\\ S_{1}=0\;or\;u=0\text{,}\\ P_{1}=0\;or\;w^{\prime }=0\text{,}\\ P_{1}^{\prime }=0\;or\;w=0\text{.} \end{array}$$

### 4.6 Final governing equations based on NSGT

To derive constitutive equations based on stress resultants, consider the NSGT stress-strain relation as

(29) $$\left[1-{\left(ea\right)}^{2}\nabla^{2}\right]{\tilde{t}}_{xx}=E\left(1-l^{2}\nabla^{2}\right)\varepsilon_{xx}\text{.}$$

By substituting Eq. (2) into Eq. (29) and integrating based on stress resultant definitions (Eq. (17) and (23)), the axial force of the bulk and the surface (N and $N^{s}$ respectively) is obtained as,

(30) $$N-{\left(ea\right)}^{2}\frac{\partial^{2}N}{\partial x^{2}}=EA\left(1-l^{2}\frac{\partial^{2}}{\partial x^{2}}\right)\frac{\partial u}{\partial x}\text{,}$$

(31) $$N^{s}-{\left(ea\right)}^{2}\frac{\partial^{2}N^{s}}{\partial x^{2}}=\left(1-l^{2}\frac{\partial^{2}}{\partial x^{2}}\right)A_{s}\frac{\partial u}{\partial x}\text{,}$$

where

(32) $$A_{s}=\int_{-h/2}^{h/2}\left(2\mu^{s}+\lambda^{s}\right)ds\text{.}$$

Similarly, the bending moment of the bulk and surface layer is obtained by considering Eqs. (17), (23) and (29) as

(33) $$M-{\left(ea\right)}^{2}\frac{\partial^{2}M}{\partial x^{2}}=EI\left(1-l^{2}\frac{\partial^{2}}{\partial x^{2}}\right)\left(-\frac{\partial^{2}w}{\partial x^{2}}\right)\text{,}$$

(34) $$M^{s}-{\left(ea\right)}^{2}\frac{\partial^{2}M^{s}}{\partial x^{2}}=\left(1-l^{2}\frac{\partial^{2}}{\partial x^{2}}\right)\left(-B_{s}\frac{\partial^{2}w}{\partial x^{2}}\right)\text{,}$$

where $B_{s}$ and I are defined as follows

(35) $$B_{s}=\int_{-h/2}^{h/2}\left(2\mu^{s}+\lambda^{s}\right)z^{2}\;ds,I=\int_{A}z^{2}\;dA\text{.}$$

Finally, the first and second non-classical equations of motion result as,

(36) $$\begin{array}{l} -EA\left(1-l^{2}\frac{\partial^{2}}{\partial x^{2}}\right)\frac{\partial^{2}u}{\partial x^{2}}-\left(1-l^{2}\frac{\partial^{2}}{\partial x^{2}}\right)\left(A_{s}\frac{\partial^{2}u}{\partial x^{2}}\right)-\left(1-{\left(ea\right)}^{2}\frac{\partial^{2}}{\partial x^{2}}\right)\left(\frac{\partial S_{1}}{\partial x}\right)+\\ \left(1-{\left(ea\right)}^{2}\frac{\partial^{2}}{\partial x^{2}}\right)(\left[\rho bh+2\rho^{s}\left(b+h\right)\right]\left(\frac{\partial^{2}u}{\partial t^{2}}\right)+m\frac{\partial^{2}u}{\partial t^{2}}\bar{\delta }\left(x-x_{m}\right)+\\ m\frac{\partial u}{\partial t}\frac{\partial }{\partial t}\bar{\delta }\left(x-x_{m}\right)+mV_{m}\frac{\partial }{\partial t}\bar{\delta }\left(x-x_{m}\right))=0\text{,} \end{array}$$

(37) $$\begin{array}{l} -EI\left(1-l^{2}\frac{\partial^{2}}{\partial x^{2}}\right)\left(-\frac{\partial^{4}w}{\partial x^{4}}\right)-\left(1-{\left(ea\right)}^{2}\frac{\partial^{2}}{\partial x^{2}}\right)\left(\frac{\partial^{2}P_{1}}{\partial x^{2}}\right)+\left(1-l^{2}\frac{\partial^{2}}{\partial x^{2}}\right)\left(B_{s}\frac{\partial^{4}w}{\partial x^{4}}\right)+\\ \left(1-{\left(ea\right)}^{2}\frac{\partial^{2}}{\partial x^{2}}\right)(mg\bar{\delta }\left(x-x_{m}\right)+\left[\rho bh+2\rho^{s}\left(b+h\right)\right]\left(\frac{\partial^{2}w}{\partial t^{2}}\right)+\\ m\frac{\partial^{2}w}{\partial t^{2}}\bar{\delta }\left(x-x_{m}\right)+m\frac{\partial w}{\partial t}\frac{\partial }{\partial t}\bar{\delta }\left(x-x_{m}\right)+2mV_{m}\frac{\partial^{2}w}{\partial x\partial t}\bar{\delta }\left(x-x_{m}\right)+\\ mV_{m}\frac{\partial w}{\partial t}\frac{\partial }{\partial x}\bar{\delta }\left(x-x_{m}\right)+mV_{m}\frac{\partial w}{\partial x}\frac{\partial }{\partial t}\bar{\delta }\left(x-x_{m}\right)+K_{w}bw-K_{G}b\frac{\partial^{2}w}{\partial x^{2}})=0\text{.} \end{array}$$

## 5 Dimensionless ODE equations

Considering the boundary conditions (Eq. (28)), displacement components can be written as [31].

(38) $$u\left(x,t\right)=\hat{u}\left(t\right)\sin\left(\frac{\pi x}{L}\right),w\left(x,t\right)=\hat{w}\left(t\right)\sin\left(\frac{\pi x}{L}\right)\text{.}$$

Substituting Eq. (38) into Eqs. (36), (37) and implying the Galerkin method, results in ODE equations as

(39) $$\begin{array}{l} (\frac{1}{2}\pi^{4}\Phi^{2}\lambda^{2}R_{1}+\pi^{4}\Phi^{2}\varsigma \lambda^{2}+\pi^{4}\Phi^{2}\lambda^{2}+\frac{1}{2}\pi^{2}\Phi^{2}R_{1}+\pi^{2}\Phi^{2}\varsigma +\pi^{2}\Phi^{2}-\\ \zeta \pi^{2}\Phi^{2}\left(\frac{1}{2}+\frac{1}{2}\cos\left(2\tau \right)\right)M+\zeta \pi^{2}\Phi^{2}M)\frac{d^{2}\bar{u}}{d\tau^{2}}+\zeta \pi^{2}\Phi^{2}M\;\sin\left(2\tau \right)\frac{d\bar{u}}{d\tau }+\\ \frac{1}{2}\left(\frac{\pi^{4}\bar{A}\gamma^{2}}{\zeta }+\frac{\pi^{4}a_{s}\gamma^{2}}{\zeta^{3}}+\zeta \pi^{2}\bar{A}+\frac{\pi^{2}a_{s}}{\zeta }\right)\bar{u}+\zeta \pi M\Phi^{2}\;\cos\;\tau =0\text{,} \end{array}$$

(40) $$\begin{array}{l} (\frac{-i\pi^{6}\Phi^{2}\lambda^{2}\upsilon }{\zeta^{3}}+\frac{\pi^{4}\Phi^{2}\varsigma \lambda^{2}}{\zeta }+\frac{\pi^{4}\Phi^{2}\lambda^{2}R_{1}}{2\zeta }-\pi^{4}\Phi^{2}\left(\frac{1}{2}+\frac{1}{2}\cos\left(2\tau \right)\right)\lambda^{2}M-\frac{i\pi^{4}\Phi^{2}\upsilon }{\zeta^{3}}+\\ \pi^{2}\Phi^{2}M+\frac{\pi^{2}\Phi^{2}}{\zeta }+\frac{\pi^{2}\Phi^{2}R_{1}}{2\zeta }-\pi^{2}\Phi^{2}\left(\frac{1}{2}+\frac{1}{2}\cos\left(2\tau \right)\right)M+\frac{\pi^{4}\Phi^{2}\lambda^{2}}{\zeta }+\pi^{4}\Phi^{2}\lambda^{2}M+\\ \frac{\pi^{2}\Phi^{2}\varsigma }{\zeta })\frac{d^{2}\bar{w}}{d\tau^{2}}+\left(\pi^{4}\;\sin\left(2\tau \right)\Phi^{2}\lambda^{2}M+\pi^{2}\;\sin\left(2\tau \right)\Phi^{2}M\right)\frac{d\bar{w}}{d\tau }+(-\frac{i\pi^{6}{\bar{\tau }}_{s}\lambda^{2}\upsilon }{\zeta^{4}}+\frac{\pi^{4}k_{G}\varsigma \lambda^{2}}{2\zeta^{2}}-\\ \frac{i\pi^{4}{\bar{\tau }}_{s}\upsilon }{\zeta^{4}}+6\pi^{4}\Phi^{2}\left(\frac{1}{2}+\frac{1}{2}\cos\left(2\tau \right)\right)\lambda^{2}M)+\frac{1}{2}\frac{i\pi^{4}}{\zeta^{4}}-3\pi^{2}\Phi^{2}M+\frac{\pi^{4}b_{s}}{2\zeta^{4}}-\\ 3\pi^{4}\Phi^{2}\lambda^{2}M+6\pi^{2}\Phi^{2}M\left(\frac{1}{2}+\frac{1}{2}\cos\left(2\tau \right)\right)+\frac{1}{2}\pi^{2}k_{w}\varsigma \lambda^{2}+\\ \frac{1}{2}\frac{\pi^{2}k_{G}\varsigma }{\zeta^{2}}+\frac{1}{2}\frac{\pi^{6}\gamma^{2}b_{s}}{\zeta^{6}}+\frac{1}{2}\frac{i\pi^{6}\gamma^{2}}{\zeta^{6}}+\frac{1}{2}k_{w}\varsigma )\bar{w}+\zeta R_{2}M\left(1+\pi^{2}\lambda^{2}\right)\sin\;\tau =0\text{,} \end{array}$$

in which the following definitions have been utilized.

(41) $$\begin{array}{l} \tau =\frac{\pi V_{m}\;t}{L},X=\frac{x}{L},\bar{u}\left(\tau \right)=\frac{\hat{u}\left(t\right)}{L},\bar{w}\left(\tau \right)=\frac{\hat{w}\left(t\right)}{h},\lambda =\frac{ea}{L},\Phi^{2}=\frac{V_{m}^{2}\rho^{s}}{EL},M=\frac{m}{\rho_{s}L^{2}}\text{,}\\ \zeta =\frac{L}{h},\varsigma =\frac{b}{h},a_{s}=\frac{A_{s}}{Eh^{2}},\bar{A}=\frac{A}{L^{2}},\gamma =\frac{l}{h},k_{w}=\frac{K_{w}h}{E},k_{G}=\frac{K_{G}}{Eh},i=\frac{I}{h^{4}}\text{,}\\ b_{s}=\frac{B_{s}}{Eh^{4}},{\bar{\tau }}_{s}=\frac{\tau_{s}}{Eh},R_{2}=\frac{\rho^{s}g}{E},R_{1}=\frac{\rho \;b}{\rho^{s}}\text{.} \end{array}$$

## 6 IHBM

The IHBM is an effective method for the analysis of dynamic stability of systems. In this method, a specific point on the instability boundary is assumed, and then, through an incremental process, additional nearby points are determined.

(42) $$\bar{u}\left(\tau \right)={\bar{u}}^{*}\left(\tau \right)+\Delta \bar{u}\left(\tau \right),\bar{w}\left(\tau \right)={\bar{w}}^{*}\left(\tau \right)+\Delta \bar{w}\left(\tau \right),M=M^{*}+\Delta M,\Phi =\Phi^{*}+\Delta \Phi \text{.}$$

Substituting Eq. (42) into Eqs. (39), (40) and eliminating nonlinear elements, a linear matrix equation is obtained in terms of incremental terms.

(43) $$\begin{array}{l} \Phi^{*2}\left(M^{*}M^{\prime }\left(\tau \right)+M^{\prime \prime }\right)\Delta \ddot{q}+M^{*}{\Phi^{*}}^{2}C\left(\tau \right)\;\Delta \dot{q}+K\left(\tau \right)\Delta q=R-\Phi^{*2}(M^{\prime }\left(\tau \right){\ddot{q}}^{*}+C\left(\tau \right){\dot{q}}^{*}+\\ K^{\prime }\left(\tau \right)Q^{*})\Delta M-2\Phi^{*}\left(\left(M^{*}M^{\prime }\left(\tau \right)+M^{\prime \prime }\right){\ddot{q}}^{*}+M^{*}C\left(\tau \right)\;{\dot{q}}^{*}+M^{*}\left(K^{\prime }\left(\tau \right)\;q^{*}\right)\right)\Delta \Phi \text{,} \end{array}$$

where

(44) $$\begin{array}{l} M^{\prime }=\left[\begin{array}{cc} m_{11}^{\prime } & 0\\ 0 & m_{22}^{\prime } \end{array}\right],M^{\prime \prime }=\left[\begin{array}{cc} m_{11}^{\prime \prime } & 0\\ 0 & m_{22}^{\prime \prime } \end{array}\right],C=\left[\begin{array}{cc} C_{11} & 0\\ 0 & C_{22} \end{array}\right],K=\left[\begin{array}{cc} K_{11} & 0\\ 0 & K_{22} \end{array}\right]\text{,}\\ K^{\prime }=\left[\begin{array}{cc} 0 & 0\\ 0 & K_{22}^{\prime } \end{array}\right],q^{*}={\left[\begin{array}{cc} {\bar{u}}^{*} & {\bar{w}}^{*} \end{array}\right]}^{T},\Delta q={\left[\begin{array}{cc} \Delta \bar{u} & \Delta \bar{w} \end{array}\right]}^{T}\text{,} \end{array}$$

and the components of the above matrices have been stated in the Appendix. R is the corrective term and this parameter is zero on the transition curve.

(45) $$R=-\left[\Phi^{*2}\left(M^{*}M^{\prime }\left(\tau \right)+M^{\prime \prime }\right){\ddot{q}}^{*}+M^{*}{\Phi^{*}}^{2}C\left(\tau \right)\;{\dot{q}}^{*}+K\left(\tau \right)q^{*}\right]$$

It should be noted that derivation concerning τ has been shown with dot superscript in Eq. (43).

**Fig. 2:**
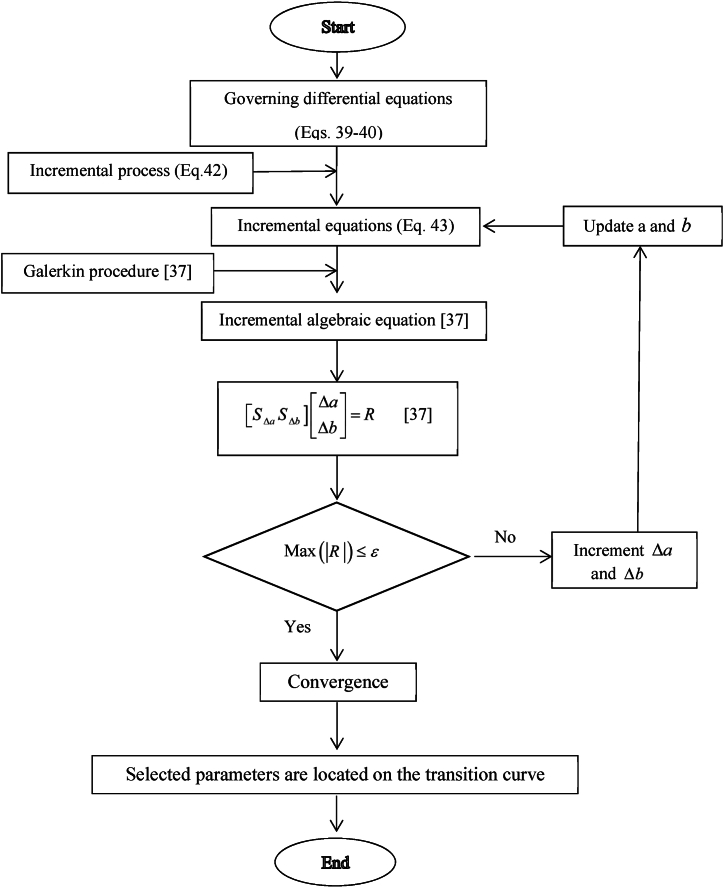
IHBM algorithm.

IHBM utilizes Fourier series with unknown coefficients $\left(a_{i},b_{i}\right)$ for determining the DIR considering T and 2T responses [37]. The IHBM algorithm can be shown in Fig. 2.

## 7 Results and discussion

The properties of the silicon nanobeam and Pasternak foundation are listed in Table 1.

**Table 1:** Properties of silicon nanobeam [31,38].

L=13(nm)	b=h=1.3(nm)	$\rho^{s}=3.17\times 10^{-7}\;\left(kg/m^{2}\right)$
$E^{s}=-10.6543\left(N/m\right)$	υ=0.24	E=210(GPa)
$\rho =2331\left(kg/m^{3}\right)$	$\tau^{s}=0.605\left(N/m\right)$	αx=−1.6(1/K∘)
$K_{w}=8.9995035\times 10^{17}\left(N/m^{3}\right)$	$K_{G}=2.071273\;\left(N/m\right)$	l=ea=1(nm)

**Fig. 3:**
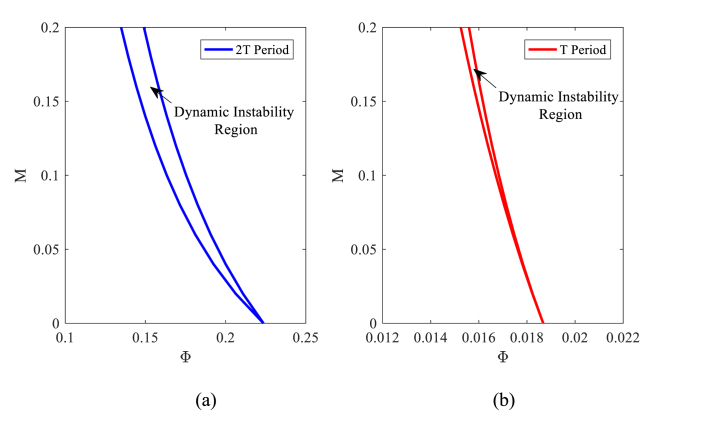
Nanobeam DIRs for (a) period 2T and (b) period T.

DIRs of nanobeam based on IHBM are displayed in Fig. 3(a) and (b) for T and 2T periods, respectively.

**Fig. 4:**
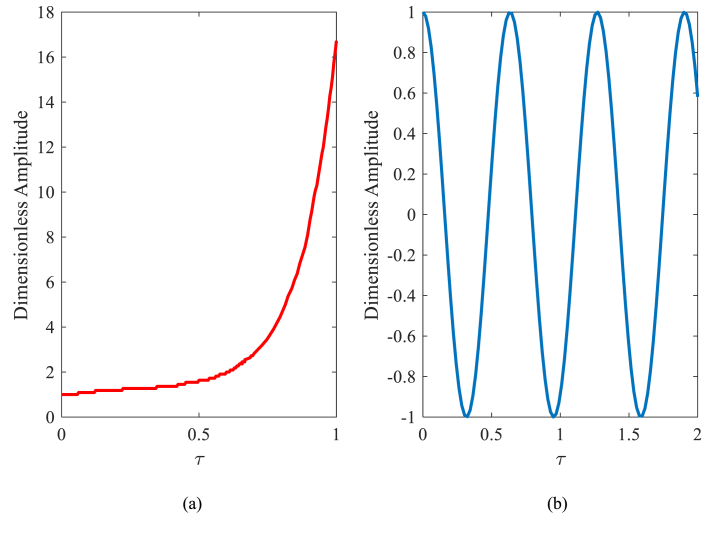
Time response diagrams: (a) unstable point (M=0.15,Φ=0.016) (b) stable point (M=0.15,Φ=0.015) in DIRs of nanobeam.

Dynamic stability diagrams are drawn in nanoparticle mass coordinates in terms of nanoparticle velocity. The mass and velocity of the nanoparticles are dimensionless. As indicated in the figure, regions of instability occurred between the two tabs. The dynamic instability regions occurred in the T period due to lower velocities of nanoparticles compared to the 2T period. And because of this, the nanobeam has been more stable in the 2T period. Furthermore, the width of unstable areas in the T period was much less in comparison to the 2T period. That's the reason why texts focusing on the 2T period gain more interest [39]. To evaluate the results and consider that the dynamic stability diagrams were not drawn in the particle mass-velocity coordinates in the literature, the time history of the first mode vibration was drawn in Fig. 4(a) and (b). In Fig. 4(a), the stability of the point M=0.15 and Φ=0.016 located in the unstable region in the T period has been checked by the Runge-Kutta method. As expected, the chart has diverged and this shows instability. In contrast, the point M=0.15 Φ=0.015 is selected within the stable region, and as shown in the time response diagram, this point is stable (Fig. 4(b)).

**Fig. 5:**
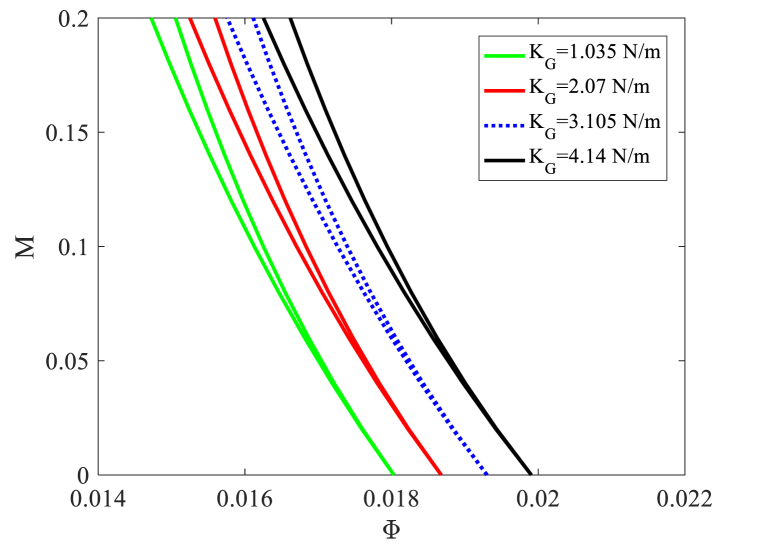
The effect of the Pasternak shear constant on DIRs of nanobeam for T periods.

**Fig. 6:**
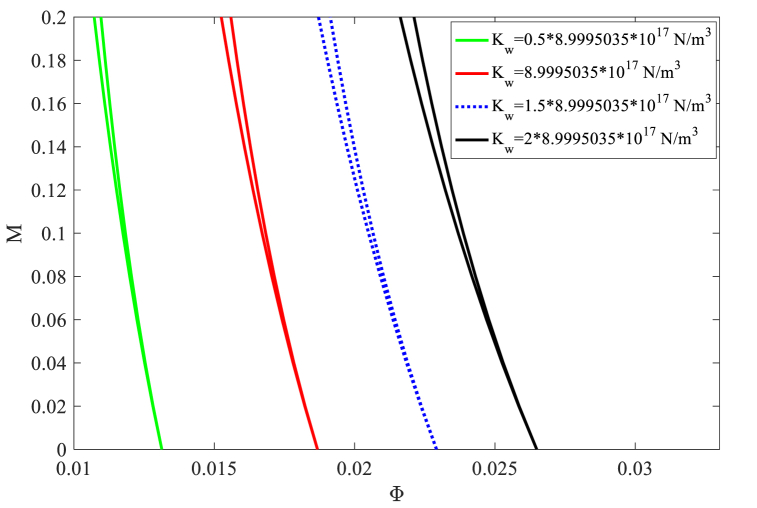
The effect of Winkler spring constant on DIRs of nanobeam for T periods.

Fig. 5, Fig. 6 show the effects of Pasternak foundation constants on DIR. Fig. 5 illustrates how the DIR of the nanobeam is affected by the Pasternak shear constant. As the Pasternak shear constant increases, the DIR shifts towards higher velocities. When the foundation becomes stiffer, the stiffness of the nanobeam increases, resulting in improved stability. Increasing $K_{G}$ from 1.035 to 4.14 (N/m) moves the origin of the DIR towards a stable region by 10 %.

Fig. 6, displays how nanobeam's DIR is affected by the Winkler spring constant. According to this figure, enhancement of the Winkler spring constant shifts the DIR towards the more stable zone. Despite the slight increase in the width of the instability zone, the origin is shifted towards higher velocities by $K_{w}$ growth. This figure indicates that increasing the $K_{w}$ from $0.5\times 8.9995035\times 10^{17}$ to $2\times 8.9995035\times 10^{17}\;\left(N/m^{3}\right)$ shifts the origin of the DIR towards higher velocities by 102 %. In other words, the Winkler spring constant is more effective for DIR movement in contrast to the Pasternak shear constant.

**Fig. 7:**
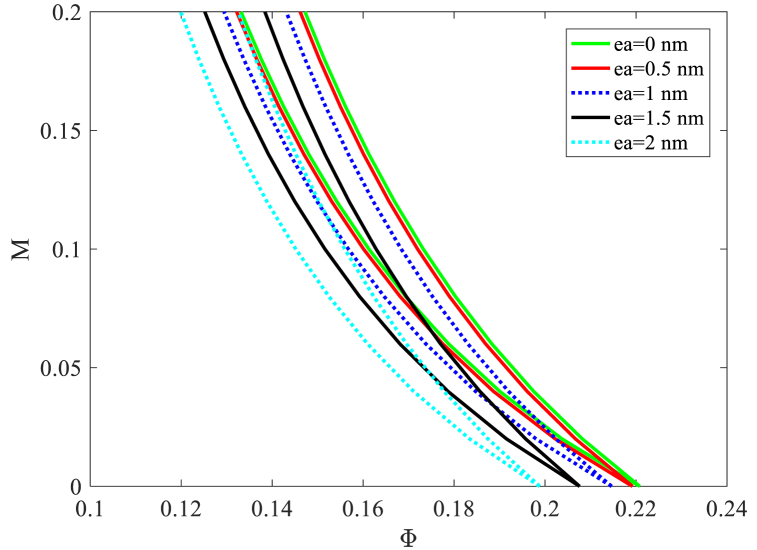
The effect of small scale parameter on nanobeam DIRs for 2T periods.

The influence of the small-scale parameter on nanobeam DIRs for the 2T period is displayed in Fig. 7. As seen in this figure, the small-scale parameter increase displaces the nanobeam DIRs towards lower velocities. So, the nanobeam gets to be more unstable by expanding the small-scale parameter. By expanding this parameter, which speaks to the bond length between particles, the nanobeam rigidity diminishes and results in lower stability. Based on this figure, the classical model predicts more stability in comparison to the nonlocal model. Increasing the nonlocal parameter 2nm shifts the origin of the nanobeam DIR towards lower velocities by 9.3 %.

**Fig. 8:**
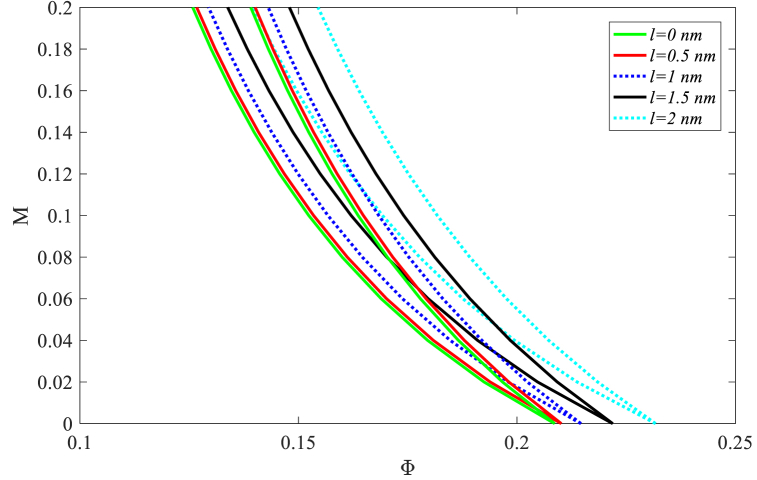
The effect of material length scale parameter on nanobeam DIRs for 2T periods.

Finally, Fig. 8 illustrates the impact of the material length scale parameter on the nanobeam DIR for a 2T period. As expected, with the increase of this parameter, the nanobeam becomes more stable. Increasing the material length scale parameter related to the strain gradient hypothesis increases the rigidity by increasing the flexural stiffness, thereby it increases the nanobeam stiffness and making it more stable. Increasing the material length scale parameter from 0.5 to 2nm shifts the origin of the nanobeam DIR by 10.3 % towards the higher velocities. In other words, material length scale and nonlocal parameters exhibit opposite effects on DIRs of nanobeams under the sequence of moving nanoparticles. So, softening and stiffening effects result from increasing nonlocal and material length scale parameters based on the direction of the DIR movement.

## 8 Conclusion

This investigation focused on the dynamic stability of an EBT nanobeam subjected to a sequence of moving inertial nanoparticles based on NSGT. At each time, a nanoparticle is in motion on the beam. The simply supported nanobeam rests on the Pasternak foundation. The governing equations, which took into account surface effects, were obtained by using Hamilton's principle. IHBM was implemented to determine nanobeam DIRs on the plane of mass versus velocity of the nanoparticle. Finally, the effects of different parameters were discussed on the nanobeam DIRs. Results show:

• Pasternak shear constant shifts the nanobeam DIR towards higher velocities. If this parameter is increased from 1.035 to 4.14 (N/m), the DIR's origin will move 10 % closer to the higher velocity region.
• By enhancing the Winkler spring constant, the DIR is shifted towards a more stable zone. Although the width of the instability zone increases slightly, the origin is moved towards higher velocities due to the growth of $K_{w}$. This indicates that increasing $K_{w}$ from $0.5\times 8.9995035\times 10^{17}$ to $2\times 8.9995035\times 10^{17}\;\left(N/m^{3}\right)$ results in a 102 % shift of the DIR's origin towards higher velocities.
• If the nonlocal parameter is increased from 0.5 to 2(nm), the origin of the nanobeam DIR will be shifted towards lower velocities by 9.3 %.
• Increasing the material length scale parameter from 0.5 to 2(nm) results in the origin movement of the DIR towards a higher velocity zone by 10.3 %.

## CRediT authorship contribution statement

**Mohammad Hashemian:** Data curation, Conceptualization, Data curation, Conceptualization. **Dheyaa J. Jasim:** Supervision, Software, Supervision, Software. **S. Mohammad Sajadi:** Formal analysis, Data curation, Conceptualization, Formal analysis, Data curation, Conceptualization. **Rahman Khanahmadi:** Visualization, Validation. **Mostafa Pirmoradian:** Visualization, Project administration, Methodology, Visualization, Project administration, Methodology. **Soheil Salahshour:** Formal analysis, Data curation, Conceptualization, Software, Methodology, Conceptualization, Software, Methodology, Conceptualization.

## Declaration of competing interest

The authors declare that they have no known competing financial interests or personal relationships that could have appeared to influence the work reported in this paper.

## Appendix

$$\begin{array}{l} m_{11}^{\prime }=-\frac{1}{2}\zeta \pi^{2}-\frac{1}{2}\zeta \pi^{2}\;\cos\left(2\tau \right)+\zeta \pi^{2}\text{,}\\ m_{22}^{\prime }=-\pi^{4}\left(\frac{1}{2}+\frac{1}{2}\cos\left(2\tau \right)\right)\lambda^{2}+\pi^{2}-\pi^{2}\left(\frac{1}{2}+\frac{1}{2}\cos\left(2\tau \right)\right)+\pi^{4}\lambda^{2}\text{,}\\ m_{11}^{\prime \prime }=\frac{1}{2}\pi^{4}\lambda^{2}R_{1}+\pi^{4}\varsigma \lambda^{2}+\pi^{4}\lambda^{2}+\frac{1}{2}\pi^{2}R_{1}+\pi^{2}\varsigma +\pi^{2}\text{,} \end{array}$$

$$m_{22}^{\prime \prime }=\frac{-i\pi^{6}\lambda^{2}\upsilon }{\zeta^{3}}+\frac{\pi^{4}\varsigma \lambda^{2}}{\zeta }+\frac{\pi^{4}\lambda^{2}R_{1}}{2\zeta }-\frac{i\pi^{4}\upsilon }{\zeta^{3}}+\frac{\pi^{2}}{\zeta }+\frac{\pi^{2}R_{1}}{2\zeta }+\frac{\pi^{4}\lambda^{2}}{\zeta }+\frac{\pi^{2}\varsigma }{\zeta }\text{,}$$

$$C_{11}=\zeta \pi^{2}\;\sin\left(2\tau \right)\text{,}$$

$$C_{22}=\pi^{4}\;\sin\left(2\tau \right)\lambda^{2}+\pi^{2}\;\sin\left(2\tau \right)\text{,}$$

$$K_{11}=\frac{1}{2}\left(\frac{\pi^{4}\bar{A}\gamma^{2}}{\zeta }+\frac{\pi^{4}a_{s}\gamma^{2}}{\zeta^{3}}+\zeta \pi^{2}\bar{A}+\frac{\pi^{2}a_{s}}{\zeta }\right)\text{,}$$

$$\begin{array}{l} K_{22}=-\frac{i\pi^{6}{\bar{\tau }}_{s}\lambda^{2}\upsilon }{\zeta^{4}}+\frac{\pi^{4}k_{G}\varsigma \lambda^{2}}{2\zeta^{2}}-\frac{i\pi^{4}{\bar{\tau }}_{s}\upsilon }{\zeta^{4}}+6\pi^{4}\Phi^{2}\left(\frac{1}{2}+\frac{1}{2}\cos\left(2\tau \right)\right)\lambda^{2}M)+\frac{1}{2}\frac{i\pi^{4}}{\zeta^{4}}-3\pi^{2}\Phi^{2}M+\\ \frac{\pi^{4}b_{s}}{2\zeta^{4}}-3\pi^{4}\Phi^{2}\lambda^{2}M+6\pi^{2}\Phi^{2}M\left(\frac{1}{2}+\frac{1}{2}\cos\left(2\tau \right)\right)+\frac{1}{2}\pi^{2}k_{w}\varsigma \lambda^{2}+\frac{1}{2}\frac{\pi^{2}k_{G}\varsigma }{\zeta^{2}}+\frac{1}{2}\frac{\pi^{6}\gamma^{2}b_{s}}{\zeta^{6}}+\\ \frac{1}{2}\frac{i\pi^{6}\gamma^{2}}{\zeta^{6}}+\frac{1}{2}k_{w}\varsigma \text{,} \end{array}$$

$$K_{22}^{\prime }=6\pi^{4}\lambda^{2}\left(\frac{1}{2}+\frac{1}{2}\cos\left(2\tau \right)\right)-3\pi^{2}-3\pi^{4}\lambda^{2}+6\pi^{2}\left(\frac{1}{2}+\frac{1}{2}\cos\left(2\tau \right)\right)\text{.}$$
